# Aligned bodies, united hearts: embodied emotional dynamics of an Islamic ritual

**DOI:** 10.1098/rstb.2023.0162

**Published:** 2024-10-07

**Authors:** Mohammadamin Saraei, Alexandra Paxton, Dimitris Xygalatas

**Affiliations:** ^1^ Department of Psychological Sciences, University of Connecticut, Storrs, CT, USA; ^2^ Center for the Ecological Study of Perception and Action, University of Connecticut, Storrs, CT, USA; ^3^ Department of Anthropology, University of Connecticut, Storrs, CT, USA

**Keywords:** embodiment, synchrony, ritual, Salat al Jama’ah, prayer

## Abstract

Collective rituals involve the coordination of intentions and actions and have been shown to promote the alignment of emotional states and social identities. However, the mechanics of achieving group-level synchrony is yet unclear. We report the results of a naturalistic study in the context of an Islamic congregational prayer that involves synchronous movement. We used wearable devices to capture data on body posture, autonomic responses and spatial proximity to investigate how postural alignment and shared arousal intertwine during this ritual. The findings reveal a dual process at play: postural alignment appears to be more localized, with worshippers synchronizing their movements with their nearest neighbours, while physiological alignment operates on a broader scale, primarily driven by the central role of the religious leader. Our findings underscore the importance of interpersonal dynamics in collective gatherings and the role of physical co-presence in fostering connections among participants, with implications extending to our understanding of group dynamics across various social settings.

This article is part of the theme issue ‘Minds in movement: embodied cognition in the age of artificial intelligence’.

## 1. Introduction

Ritualized collective gatherings play crucial roles in all human cultures. By promoting social coordination, group cohesion and norm adherence, they contribute to the maintenance of social order within a given group [1]. Indeed, social theorists have argued that communal participation in such gatherings may have acted as a vehicle for the establishment of the first human societies [2–4]. A variety of features may contribute to these effects, from the brandishing of symbolic markers and appeals to tradition, to the use of sensory extravagance and the formation of shared memories [5]. Key among these features is the ability of ritualized collective action to produce emotional alignment through the embodied experience of shared participation. Rather than merely symbolic, the physical actions involved in ritual practice are integral to that experience [6].

A common aspect of these ritual activities across diverse cultures is synchrony or engaging in similar behaviours in time with others [7]. Various perspectives have emphasized the ability to engage in synchronous actions such as chorusing [8,9], and although synchrony can emerge unintentionally, synchrony in these settings is often important to the performance of the ritual. Synchrony has been shown to have potential effects at various levels, including behaviour, perception, cognition and emotion [10], and to enhance feelings of belonging and self-transcendence in the context of rituals [11,12].

Previous research has documented the spontaneous synchronization of emotions in the context of public events [7], and such synchrony has been linked to group cohesion [8]. Importantly, these effects are believed to have a social basis. For example, people are more likely to mirror the emotional reactions of socially relevant individuals [9] and are more likely to do so in real life rather than virtual contexts [8]. Unsurprisingly, then, collective rituals commonly involve the intentional coordination of behaviours through actions like dancing, chanting, marching, gesturing or bowing in synchrony. These, in turn, facilitate the alignment of emotions: when people intentionally move as one within their shared sociocultural setting, they feel like one and form stronger social connections [10–14].

However, how this group-level synchrony is achieved remains unknown. Three possibilities seem the likeliest candidates:

- does each person in the crowd attune to a single leader, like orchestra members being led by a conductor?
- does everyone synchronize with individuals nearest to them in the collective, like flocks of birds [15]? and
- does the group behave like a single unit by directly attuning to the structure of the ritual?

Moreover, the exact role of the body in facilitating physiological alignment is unclear: for instance, how does physical co-presence and proximity affect emotional coupling? To explore those questions, we conducted a naturalistic study in the context of an Islamic ritual that involves highly synchronous movement and shared intentionality, the *Salat al Jama’ah* [16].

### (a) Structure of the ritual

Various features of the *Salat al Jama’ah* [16] make it a useful setting in which to explore the relationship between shared movement and shared emotion during socioculturally important interactions. To provide the appropriate context for readers, we briefly review them here. We discuss the ritual in relation to the practices of the specific community studied in the current study, including gender-segregated prayer (according to a view of gender as binary).

**Figure 1.**
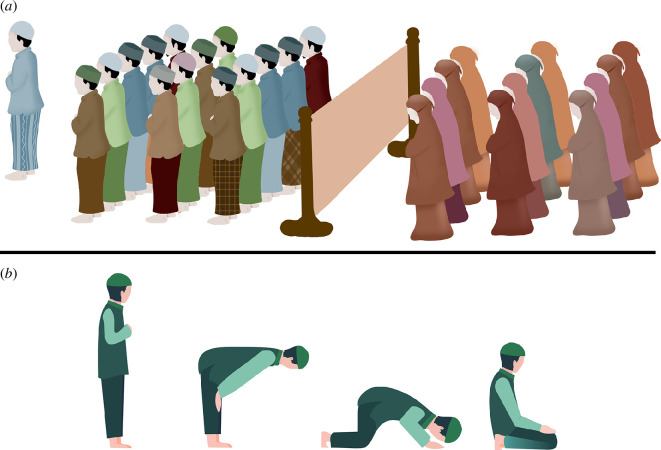
(*a*) Worshippers in the *Salat al Jama’ah* are positioned in parallel rows behind the imam, and women are separated from men by a partition or other means. (*b*) The prayer consists of repetitive units (*Rak’ah*). Each unit includes a sequence of movements followed by reciting specific parts of the prayer. In order, this movement sequence includes standing upright, bowing at a 90° angle with hands on knees, standing upright, full prostration (with the forehead, palms and big toes touching the ground) and sitting position with legs folded under the body. The *Rak’ah* is repeated multiple times in the communal prayer. Images adapted from pngtree.com.

The *Salat al Jama’ah* is a group prayer during which participants stand in parallel rows behind a religious leader (imam) while facing the same direction (i.e. qibla, which is the direction of the Kaaba in Mecca) and perform repetitive cycles of bows and prostrations while reciting prayers (see figure 1). Islamic theology places a strong emphasis on the benefits of collective performance. In the Haddith (i.e. Islamic scriptures believed to share the words of Prophet Muhammad himself), it is said that a prayer performed in congregation offers twenty-five times greater rewards than a prayer performed alone [17].

The position of the body and its relation to other bodies during this prayer is clearly and strictly prescribed [18]. Bowing must be performed by bending the body at the waist without tilting to the side and without bending the knees. During prostration, the forehead, knees, palms of both hands and both big toes must be touching the floor. Worshippers are expected to follow the body movements of the imam, who leads the prayer, but they are not expected to engage in coordinated breathing or recitation of verses. A worshipper must be located behind the imam at a distance no longer than approximately an average person’s largest footstep [18], but subsequent worshippers may be linked to the imam through another worshipper situated either in front or next to them, forming an interconnected grid. This grid should have no gaps, ensuring the equidistant placement of all worshippers in connection to their neighbours.

Based on the religious doctrine [18], all worshippers must be linked spatially to the imam or to another worshipper without visible obstructions (e.g. curtain, wall and pillar) for the prayer to be valid. Validity in this context is defined according to Islamic jurisprudence (fiqh) in accordance with sacred texts such as the Quran and Hadith [18]. According to Islamic theology, when a prayer is invalid, it is not considered as a collective prayer and needs to be repeated for any of its benefits to occur.

Note, however, that in traditional forms of this practice, such as the one we studied, women and men are separated during the prayer, and this does not invalidate the prayer. To accommodate this, women are separated from men by a partition or some other obstruction, with auditory access to the men and the imam; women perform the prayer simultaneously in an adjacent but separate space from the men and the imam. Within the group of women, the same rules of connection apply, creating a second interconnected grid of worshippers that is temporally linked but not spatially connected to the other group.

### (b) The present study

The explicit guidelines about movement and intention in the *Salat al Jama’ah* provide an opportunity to assess interpersonal alignment in a real-world setting of high community importance. Here, we use wearable monitors to collect data on behaviour (i.e. body posture), physiological arousal (i.e. heart rate (HR)) and proximity (i.e. distance in space) from both women and men, including the imam, as they engaged in this collective ritual. This allowed us to test three distinct (although not necessarily mutually exclusive) hypotheses about the nature of collective synchronization during group rituals:

- if groups synchronize as a result of being directly attuned to a central leader, we should see a leader–follower relationship between the imam and the worshippers;
- if groups synchronize as a result of individuals' local coordination, we should see greater coupling between worshippers situated in close proximity (compared to worshippers who are more spatially distinct from one another); and
- if the group synchronizes as a result of each individual’s attunement to the structure of the ritual, there would be no discernible differences between types of participants.

## 2. Methods

### (a) Participants

We recruited 28 participants (14 women, 14 men; *M*_age_ = 23.1 years; age range = 18−56 years),^1^ among a pool of roughly 250 people who participated in the prayer, according to unofficial estimates obtained from the organizers. All participants were Muslims who attended a prayer on a Thursday night (Maghrib prayer) during Ramadan in the Islamic Center of a North American University. The centre’s leadership kindly gave our team permission to conduct the study on their grounds, and the imam agreed in advance to participate in the experiment. Members of the research team approached worshippers as they arrived at the venue and offered them the opportunity to participate in the study. No specific criteria were set for participation other than being over 18 years of age. Participation in the study was voluntary and did not involve any compensation. Recruitment was as random as possible within the confines of a naturalistic study, and the researchers had no prior knowledge of where people would be positioned in space during the prayer. Owing to equipment malfunctioning, posture data from one participant and HR data from a further seven participants were excluded from the analysis. This yielded a final sample of 27 participants (14 men and 13 women) with posture measurement and 21 participants (12 men and nine women) with HR data.

To ensure participant anonymity, we intentionally refrained from collecting any identifying information. Consequently, we lack precise knowledge regarding the specific relationships between participants. However, in general, there is a mix of different social relationships among worshippers at this Islamic centre: some maintain pre-existing relationships with fellow worshippers, while others attend alone and do not have intimate acquaintances among other attendees. It is worth noting that the physical proximity observed during prayer does not necessarily stem from existing relationships but rather follows the order of arrival. Those arriving earlier tend to fill the front rows, while subsequent arrivals stand behind in successive rows.

### (b) Material and procedures

After agreeing to participate, each participant was brought to a private location and, with help of a same-gender member of the research team, was fitted with a chest strap with two attached modules: the Zephyr BioHarness 3.0 [19], which measures HR through recording of cardiac electric impulses and captures body movement through a tri-axial accelerometer and a SafeSpacer [20], a proximity sensor that uses a radio signal to measure the distance between each pair of devices. The chest strap was worn under the clothes, and the devices were not visible to other observers.

After the sensors were checked, participants went to the prayer room, where they performed their usual activities. Those included waiting for the call to prayer (Adhan), participating in the prayer as described above and finally moving to a hall room to dine and socialize. At the end of the event, each participant returned to the research team when they were ready to leave the venue and answered a few questions about their experience in the prayer. They then returned the devices and left the centre. Data were recorded locally on each device and downloaded to a computer after the session.

### (c) Data preparation and analysis

For the current study, we considered data only from the prayer, which lasted approximately 6 min. HR data, sampled at 1 Hz, were processed in R (v. 4.2.2) [21]. Posture data were also sampled at 1 Hz; posture was calculated with Zephyr’s proprietary algorithm, which provides a single measure of posture measured in degrees (±180) from a vertical position. Statistical analyses were carried out in Jamovi [22].

Data collected from wearable devices often include missing values [23]. Analyses suggest that it is unproblematic to use simple imputation techniques when less than 10% of the data are missing [24]. In our dataset, 0.005% of data points were missing from the HR data and were filled with series mean imputation.

We used cross-recurrence quantification analysis (CRQA) to assess synchrony or the temporal coupling of two signals [25]. CRQA is a dynamical analysis used to quantify similarities between two signals as they unfold over time [7,26]. Unlike linear analyses like cross-correlation, nonlinear approaches like CRQA can capture both linear and nonlinear patterns in the data, allowing a fuller modelling of complex physiological phenomena [27].

Excellent introductions to nonlinear analyses are available elsewhere [28,29], but we here provide a brief overview of the process for those who are unfamiliar with the technique. Continuous CRQA starts by embedding each time series into a multidimensional phase space through a process of phase-space reconstruction. This allows a full unfolding of each of the two interacting systems that we can use to identify all instances when two-time series move through similar regions of the phase space. We can plot these co-visitations of similar states (or recurrences) in a matrix (or cross-recurrence plot, CRP), which can be quantified through various metrics. In the current work, we focus on two: per cent recurrence (%REC), which we can think of as the overall proportion of shared states across the CRP; and per cent determinism (%DET), which we can think of as the reliability of the coupling across the CRP. REC is commonly interpreted as the inverse of noise in the new joint system [30], allowing us to see whether the two systems tend to inhabit similar states or not. DET reflects the stability and consistency of these sequences, since it measures how often these shared states are part of a longer ‘runs’ of shared states (versus non-sequential moments of sharing states). Consequently, both measures are commonly used to measure synchrony [31].

In addition to the plot-wide metrics from the CRP, we also extract a diagonal cross-recurrence profile (DCRP) [32] to investigate coupling direction in moment-to-moment interactions. In other words, DCRPs allow us to quantify leader–follower dynamics; specifically, they measure whether one time series is leading or following the other and by how much. In this analysis, instead of quantifying the entire CRP, we focus on a limited band around the line of synchrony or the main diagonal line in the CRP. This is a special case because it captures both time series at the same time, enabling us to determine whether one time series is leading the other by a specific amount of lag or not [32].

CRQA was performed on *z*-scored HR and posture data between all pairs of subjects in our sample (*n* = 351 pairs for posture and *n* = 205 pairs for HR) using the crqa package in R [33]. The delay and embedding parameters for each time series were selected by applying the average mutual information and false nearest neighbour methods [32,33] for each pair. A global radius parameter was selected to maintain a recurrence rate of 2−5%.

To determine the spatial proximity between subjects, we used SafeSpacer data to extract the mean distance in metres between the pairs of subjects during the prayer, which we corroborated with researchers’ notes on schematic drawings from the ritual. This allowed us to identify pairs that were situated adjacent to one another, which were marked as nearest neighbours. Finally, we used the R package *igraph* [34] to create a shortest path analysis and obtain a distance matrix between all pairs.

The nature of the ritual—as it is practised in this community—means that there are multiple naturally occurring groups that we can compare in order to test our questions of interest:

- by having worshippers who can see and cannot see the leader of the ritual, we can test the degree to which group-level synchronization is driven by individual synchronization to a central leader. Specifically, if group-level synchronization is driven by a central leader, we would expect to see the highest synchronization of worshippers to the imam when they are in his neighbour pairs (i.e. those who are directly behind the imam; here, exclusively men), followed by his distant pairs (i.e. those who are in the same space but who are not directly behind the imam; here, exclusively men), followed by his segregated pairs (i.e. those who are not in the same space with the imam; here, exclusively women). Although we do not have data from enough of the imam’s neighbours to analyse that group, we can here test whether distant pairs have a higher synchronization than segregated pairs;
- by having both colocated and non-colocated groups (since men and women prayed in separate spaces) with identical grid layouts within each group, we can test the degree to which group-level synchronization is driven by individuals synchronizing to local patterns versus global patterns. Thus, if group-level synchronization is more locally driven, we would expect the highest synchronization for neighbour pairs (i.e. people immediately next to one another), followed by distant pairs (i.e. people in the same space but not directly next to one another), followed by segregated pairs (i.e. people in different spaces); and
- if group-level synchronization is the result of each individual synchronizing to the shared rhythm of a familiar ritual, we would expect to see no difference in the amount of synchrony among the imam’s neighbour pairs, distant pairs and segregated pairs.

## 3. Results

Though segregated in space, men and women were seated in similar arrangements, the mean distance between nearest neighbours being 1.24 m (s.d. = 0.57) for the former and 1.22 m (s.d. = 0.51) for the latter, without any significant differences between the two (*U* = 192.50, *p* = 0.803). The maximal step-distance between individuals was equal (i.e. two steps) in both networks.

### (a) Analyses of cross-recurrence quantification analysis metrics

We used a series of 5-level factor regressions to analyse the CRQA metrics according to our predictions. Pairs of participants were classified into five non-overlapping groups:

- *imam + colocated*: pairs of the imam and a proximal worshipper (i.e. a person who was in a shared physical space; in this ritual, only men);
- *imam + non-colocated*: pairs of the imam and a separated worshipper (i.e. a person who was in a different physical space; in this ritual, only women);
- *neighbour pairs*: pairs of worshippers who were immediately physically adjacent to one another in the matrix of worshippers (in this ritual, pairs of only men or only women), with no pairs including the imam;
- *distant pairs*: pairs of worshippers who were in the same space but not immediately physically adjacent to one another (in this ritual, pairs of only men or only women), with no pairs including the imam; and
- *separated pairs (reference category)*: pairs of worshippers who were separated in space (in this ritual, only men–women pairs), with no pairs including the imam.

Looking at the posture data, we saw the highest %REC and %DET among neighbour pairs. On the other hand, the HR data only showed higher %DET for the imam + non-colocated pairs, with neighbour pairs showing a similar trend that did not reach significance (see table 1).

**Table 1.** Estimates and standard errors from linear regression models using pair categories to predict %REC and %DET from posture data and HR data. (*p < 0.5; **p < 0.01; ***p < 0.001.)

	posture		**HR**	
	%REC	%DET	%REC	%DET
(intercept)	4.52 (0.02)***	82.47 (0.14)***	2.26 (0.12)***	74.40 (1.25)***
imam + colocated	−0.14 (0.07)	−0.65 (0.52)	−0.49 (0.40)	1.45 (3.95)
imam + non-colocated	−0.06 (0.07)	−0.84 (0.52)	0.25 (0.43)	10.80 (4.33)*
neighbours	0.12 (0.04)**	0.97 (0.28)***	0.15 (0.25)	3.54 (2.53)
distant	0.03 (0.03)	−0.10 (0.23)	−0.23 (0.20)	−1.90 (2.05)

### (b) Comparison to surrogate data

To check whether the observed patterns might be simply owing to the structure of the prayer rather than the context of the event, we used Fourier phase-randomization analysis to create an artificial control group [35–37]. This technique produces a surrogate dataset by altering the phases of a signal while preserving its amplitude spectrum, thus retaining the autocorrelations of the original time series. When used in conjunction with CRQA, this technique allows us to establish a statistical baseline of the level of synchrony that might occur by chance, using the underlying properties of the observed data to create a more conservative control than other statistical baselines. (For more on phase-randomized baselines in synchrony research—including comparisons to other common baselines in this area—see [35].)

Using this method, we created 10 phase-randomized surrogate time series for each participant and re-ran the CRQA analyses using the original parameters. We then used linear mixed models with pair as a random effect to re-run the regressions by adding data type (true versus surrogate) as a predictor. Across all metrics, the original dataset was found to have higher synchrony than would be expected by chance (table 2).

**Table 2.** Estimates and standard errors from linear mixed models adjusted for the random effects of pair. (Each model used pair categories to predict %REC and %DET from posture data and HR data. Type is a dummy variable denoting true data versus surrogate data, with the latter as the reference category. *p < 0.5; **p < 0.01; ***p < 0.001.)

	posture		**HR**	
	%REC	%DET	%REC	%DET
(intercept)	0.40 (0.00)***	44.52 (0.31)***	1.70 (0.03)***	66.50 (1.01)***
imam + colocated	0.03 (0.01)*	0.75 (1.17)	0.09 (0.11)	4.93 (3.22)
imam + non-colocated	0.01 (0.01)	−2.06 (1.17).	0.34 (0.12)**	10.94 (3.53)**
neighbours	0.00 (0.01)	0.16 (0.64)	0.07 (0.07)	1.93 (2.06)
non-neighbours	0.00 (0.01)	0.01 (0.51)	0.05 (0.06)	−2.04 (1.66)
type	4.13 (0.01)***	37.95 (0.90)***	0.56 (0.05)***	8.04 (0.47)***
imam + colocated × type	−0.17 (0.05)***	−1.39 (3.36)	−0.58 (0.16)***	−3.61 (1.49)*
imam + non-colocated × type	−0.07 (0.05)	1.22 (3.36)	−0.10 (0.18)	−0.28 (1.63)
neighbours type	0.11 (0.03)***	0.81 (1.82)	0.08 (0.11)	1.47 (0.95)
non-neighbours × type	0.03 (0.02)	−0.11 (1.46)	−0.28 (0.09)**	0.00 (0.77)

### (c) Analyses of diagonal cross-recurrence profile data

**Figure 2.**
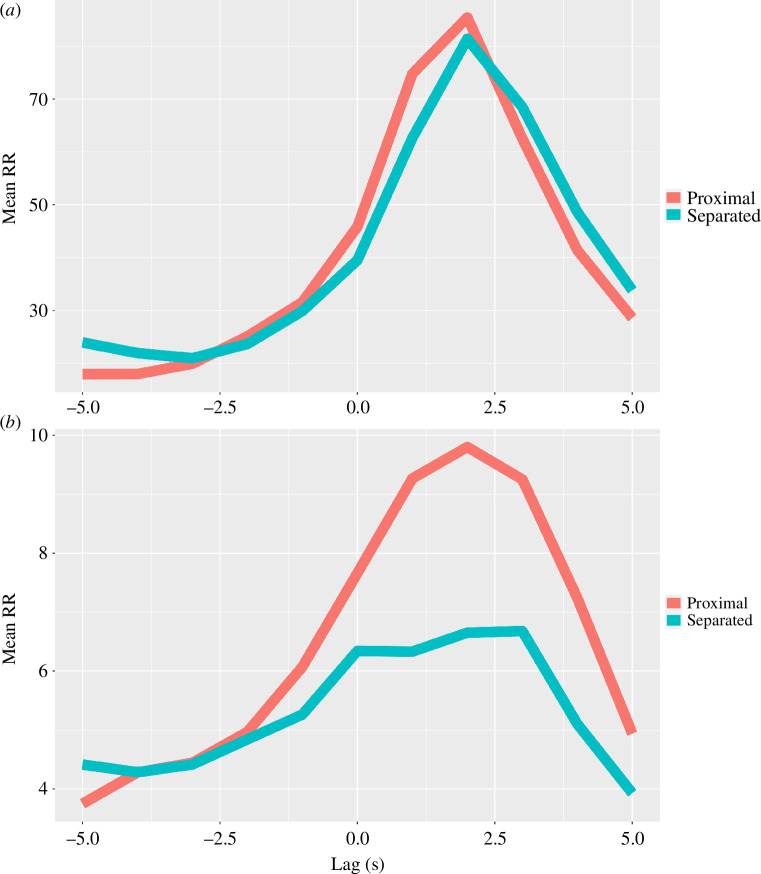
Diagonal recurrence profiles from pairwise cross-recurrence analyses of posture (*a*) and heart rate (HR) data (*b*) at various lags between the imam and the average of colocated (red) and non-colocated (blue) participants, representing the distribution of recurrent points as a percentage in a band around the line of synchronization. Here, the imam significantly leads the group in posture, as the maximum average recurrence occurs with a delay from zero; the imam also slightly leads in HR, although it does not reach statistical significance.

Analyses of the DCRP data revealed a leading dynamic for the imam, who led 96% of participants in posture (tied with one other participant for the highest lead instances), with the most common lag occurring at 2 s (mean = 1.65, s.d. = 1.50; figure 2*a*). A one-sample *t*‐test showed a significant difference from zero: *t*_25_ = 5.64, *p* < 0.001. Similarly, the imam led 70% of participants in HR (tied with one other participant for the highest lead instances), with the most common lag occurring at 1 s (mean = 0.90, s.d. = 2.43; figure 2*b*). However, a one-sample *t*‐test did not reach significance (possibly owing to the lower degrees of freedom), *t*_19_ = 1.66, *p* = 0.113.

### (d) Analyses of network data

**Figure 3.**
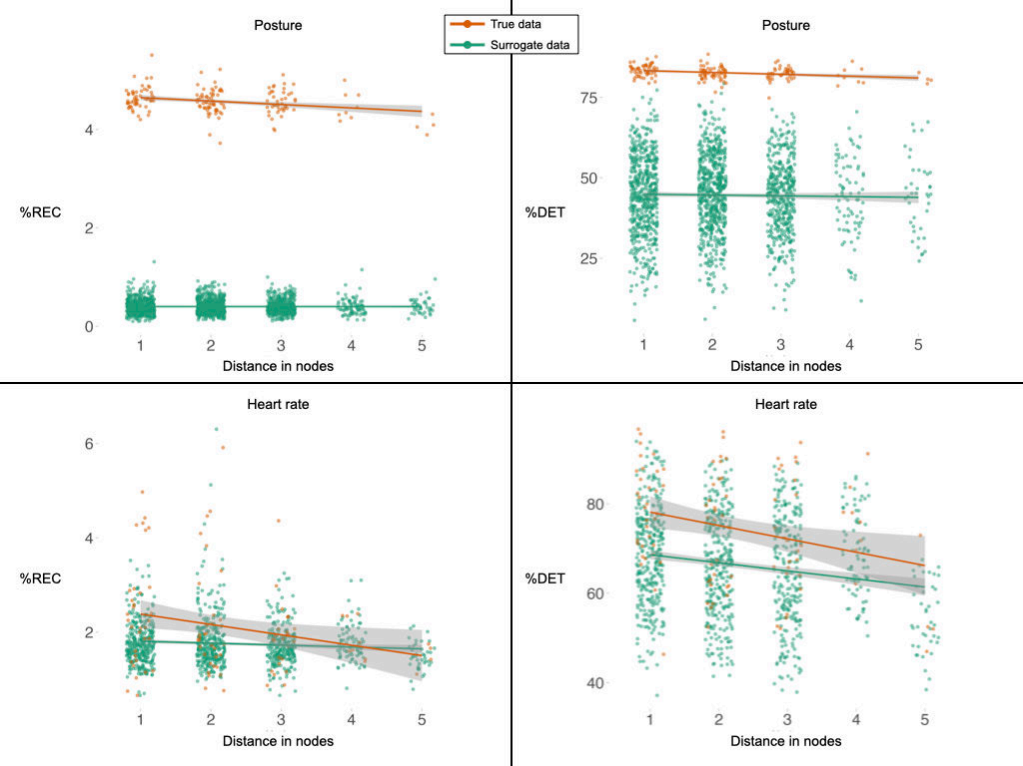
Synchrony as a function of degree of connectedness between pairs of worshippers. A value of 1 in the horizontal axis indicates that two individuals are linked directly (located within a 2 m range). A value of 2 indicates that they are linked through one intermediate link that is connected to both of them; a value of 3 indicates that they are connected through two intermediate links and so on. Error bands represent 95% confidence intervals. Overall, in the true data (orange), the closer two individuals are located in space, the more aligned their behaviours and physiology, while no such relationship is observed in the surrogate data (green).

Examinations of the network data showed that distance in space predicted the degree of synchrony across all measures. Specifically, linear regression models showed a negative relationship between distance with %REC and %DET both for the posture and the HR data. Moreover, linear mixed models confirmed that these effects were stronger in the true dataset compared with the surrogate data (table 3; figure 3).

**Table 3.** Estimates and standard errors from linear regression models (top), and linear mixed models with pair as a random factor (bottom), using pair categories to predict %REC and %DET from posture data and HR data. (*p < 0.5; **p < 0.01; ***p < 0.001.)

	posture		**HR**	
	%REC	%DET	%REC	%DET
(intercept)	4.72 (0.04)***	83.86 (0.34)***	2.60 (0.22)***	81.12 (2.70)***
nodes	−0.07 (0.02)***	−0.57 (0.15)***	−0.22 (0.09)*	−2.99 (1.10)**
	**posture**		**HR**	
(intercept)	0.40 (0.01)***	45.14 (0.78)***	1.85 (0.08)***	70.43 (2.38)***
nodes	0.00 (0.00)	−0.24 (0.34)	−0.04 (0.03)	−1.81 (0.97).
type	4.32 (0.03)***	38.72 (2.11)***	0.76 (0.10)***	10.69 (1.03)***
nodes × type	−0.07 (0.01)***	−0.33 (0.91)	−0.18 (0.04)***	−1.18 (0.42)**

### (e) Analyses of qualitative data

At the conclusion of the study, participants answered a series of self-reports about their experience and their perceived synchrony with the other worshippers. These qualitative data are summarized in table 4. All questions were worded with binary responses (i.e. allowing only ‘yes’ or ‘no’ only); all participants chose to answer all questions.

**Table 4.** Responses from participants’ post-ritual qualitative experience questionnaire. (All participants answered all questions with ‘yes’ or ‘no’ responses.)

questions	percentage of ‘yes’ answers by participants	number of participants answering ‘yes’
did you feel that your behaviour was synchronized in time with the other participants in *Salat al-Jama'ah* (congregational prayer)?	88.9	24
did you experience a feeling of togetherness when moving with the other participants in *Salat al-Jama'ah* (congregational prayer)?	92.6	25
did you feel your movements were coordinated with the other participants' movements in *Salat al-Jama'ah* (congregational prayer)?	100	27
did you feel different from the other participants?	33.3	9
did you feel similar to the other participants?	85.2	23

## 4. Discussion

The current study sought to investigate how collective rituals such as the *Salat al Jama’ah* facilitate interpersonal synchrony among participants. Our results suggest that multiple processes may be simultaneously at play, with postural alignment appearing to be more localized and physiological alignment operating on a broader scale. Posturally, worshippers synchronized their movements to their nearest neighbours, in line with previous literature on behavioural mimicry in proximal interactions [38]. Physiologically, however, the imam appeared to drive overall synchrony by creating a ripple effect in the context of the ritual, which partially supports a structured ‘leader–follower’ dynamic and reflects the central role of the imam during the prayer. In keeping with the idea of the ripple effect, both postural and physiological synchrony were highest among neighbours, highlighting the pivotal role of interpersonal dynamics in ritualistic practices. This alignment in posture and HR may have important social implications, as previous studies suggest that it might be key to social cohesion [39] by promoting trust [40], cooperation [41] and prosocial tendencies [42].

Taken together, corporeal co-presence and spatial alignment appear to facilitate social and physiological alignment within the context of shared intentionality of a community ritual. Importantly, we found that the amount of synchrony differed significantly than what we would expect by chance. This suggests that the observed effects cannot be solely attributable to a single factor, like engaging in similar sequences of movements. Rather, these patterns grow out of both the *structure* of the prayer and the *shared setting* in which it occurs. Thus, synchronization is influenced not only by the specific structure and sequence of the prayer but also by the position of other prayers, the imam and moment-by-moment interactions between them.

### (a) Limitations and future directions

While our work provides a powerful analytical case study of a real-world ritual, it has several important limitations that suggest opportunities for future work.

Second, owing to our choice of analytical methods, the current study focused on pairs of individuals. While we derived metrics of group-level dynamics through the pair-to-pair couplings, future work should consider analyses of groups *as such*—for example, through multidimensional recurrence quantification analysis [43] or coupled oscillator models [44,45].

Third, our sample was relatively small and homogeneous. We included only a single group, led by a single leader, within a relatively small community. Moreover, there were no control groups to compare with. Further analyses will benefit from a control group and a more granular exploration of individual differences. Useful potential avenues for future research on physiological and behavioral synchrony include religiosity, more standardized self-reported surveys on emotional states and perceived synchrony, familiarity with the ritual, social networks among the worshippers, socioeconomic variables, and features of the community (e.g. progressive versus traditional Islamic beliefs).

Fourth, future research may also explore the psychological and social ramifications of such synchronized events. In the current study, no data were available about pre-existing relationships among the worshippers, but those relationships could affect and be affected by their emotions and synchrony during the prayer. Although other work suggests that coordinated movement and autonomic coupling can independently contribute to social bonding [8,10–14], the pathways through which the two interact remain less known. By delving into social connections within the community, future work could shed light on these important questions.

A more in-depth examination of these mechanisms may help increase our understanding of how emotional responses spread within groups in other contexts, from sporting and musical events to public demonstrations and riots. While religiosity might influence the dynamics of this prayer, potentially enforcing strict adherence to its rules, our proposal suggests that similar circumstances, even in secular settings like a yoga class, may trigger group synchronization through the same mechanisms such as proximity and the influence of a leader or instructor. This study represents a small step towards understanding synchrony in a real-world context with deep social meeting.

### (b) Conclusion

Through a data-driven case study of Islamic group prayer, we here add to a nascent literature on embodied experiences and group synchronization during collective rituals. Worshippers’ movement and HR tended to synchronize both with the imam leading the prayer and with nearby worshippers, suggesting that collective synchronization probably emerges through attunement to both local and global dynamics; worshippers also reported very strong feelings of synchrony and togetherness after the ritual ended. Taken together, our work supports a growing perspective that the physical actions involved in collective rituals do not serve merely symbolic purposes but also actively foster social connections between individuals and emotional alignment within groups.

## Data Availability

Data are available for review at [46].

## Footnotes

^1^
Participation was limited naturally by the number of devices available for data collection.
